# Applying self-determination theory to understand maternal well-being in autism caregiving: a mini review

**DOI:** 10.3389/frcha.2026.1812579

**Published:** 2026-06-11

**Authors:** J. Kripamariya, P. J. Justin

**Affiliations:** School of Social Work, Marian College Kuttikkanam Autonomous, Kuttikkanam, India

**Keywords:** autism spectrum disorder, caregiver well-being, maternal mental health, psychological need satisfaction, self-determination theory

## Abstract

Mothers of children with autism spectrum disorder (ASD) often undergo psychological distress, care burden, and poor well-being, but current research has mostly focused on stress and deficit-based models instead of strengths-based mechanisms that facilitate well-being and resilience. Self-Determination Theory (SDT), the conceptual framework of understanding well-being because of saturation of autonomy, competence, and relatedness needs, provides a valuable conceptualization of maternal adjustment and mental wellness in caregiving situations. This mini review is a synthesis of current findings on empirical and theoretical literature on how these fundamental psychological needs are either upheld or inhibited in autism caregiving, with the focus on autonomy in decision-making, self-efficacy in caregiving, and social connectedness. It has been indicated that higher need satisfaction is always linked to better psychological health, less distress and higher caregiving confidence, whereas systemic challenges, stigma, and weak support sabotage these mechanisms. Nonetheless, very few studies formally use SDT; most of them are based on cross-sectional designs, and culturally varied populations are not well represented. Incorporating SDT into the study and intervention formulation of caregivers can help lead to empowering, need-enhancing services that encourage maternal health and child outcomes.

## 1 Introduction

Autism Spectrum Disorder (ASD) is a neurodevelopmental condition characterized by persistent difficulties in social communication and restricted, repetitive behaviors, as defined in the DSM-5-TR (1). Family members of children going through autism evaluations have shown a great deal of emotional turmoil and unease within their family system throughout this process, regardless of whether they receive a definitive diagnosis (Hickey et al. 2022). Indicatively, parents of children with ASD exhibit higher scores on the standardized measures of burden, indicating moderate-high perceived caregiving strain (2). Families that have children with autism spectrum disorder (ASD) face many emotional, social, and practical caregiving demands. Among these families, mothers typically exhibit the highest levels of parenting stress, caregiving burden, and psychological strain, which can harm family relationships and well-being (3). Previous research also supports the notion that caregiving burden and parental stress affect the quality of the parent-child relationship (Levante et al. 2025).

The relationship between maternal mental health and caregiving for a child with an autism spectrum disorder (ASD) has been examined through numerous studies. Caregiving burden includes the mental, physical, social, and financial demands of providing long-term care to a child diagnosed with an ASD (2). In addition to providing long-term supervision, caregivers must manage their child's behavior, coordinate therapies, and engage in other forms of intervention. Caregivers, primarily mothers, report that they experience more psychological distress than the general population, including elevated levels of depression, anxiety, and chronic parenting stress. Studies show that mothers of children diagnosed with an ASD tend to have poorer mental health than their nondisabled peers due to the following factors: elevated levels of ongoing demand; unresolved child behavioral difficulties; social stigma; and internalized stigma as a contributor to overall maternal stress and depression (4).

The Self-Determination Theory (SDT) provides a worthwhile model to fill this void. As the original theory, SDT is a macro-theory of human motivation and personality that highlights three fundamental psychological needs: autonomy, competence, and relatedness as key factors to achieving optimal functioning and well-being. Environments that satisfy these needs promote autonomous motivation and psychological well-being, and environments that frustrate them promote ill-being and regulated motivation according to SDT. Mothers that have a greater sense of independence in their caregiving choices (i.e., more autonomy), see themselves as capable of completing the day-to-day activities associated with being a caregiver and have sufficient social support from others may be less likely to experience symptoms of psychological distress while they are also involved in informal caregiving. Research from numerous studies on informal caregivers has shown that the more your needs are being met will equate to a greater desire to be the one to provide care for someone else, which can also lead to a better mental health experience (5, 6).

Much work has been done on caregiver stress, but not as much has been done on using Self Determination Theory (SDT) to better understand maternal mental health when caregivers are caring for someone with Autism. Research that focuses on stress is primarily focused on the burden of caregiving whereas the Self Determination Theory may also provide an answer for how caregivers can sustain their motivation, fitness, and resilience in times where they experience chronic stress and give them a strength-based approach to address social support and their identity as caregivers for mothers who are able to maintain their mental well-being or improve their mental well-being. Further, SDT is also interested in how creating an environment that supports the caregiver's basic needs to be successful aligns with the current person-centered and empowerment-based approaches to mental health and rehabilitation (7).

The four primary components of this paper, “Self-Determination Theory: A Theoretical Base for Understanding Caregiver Wellbeing,” “Current Research Gaps and Methodological Limitations,” “Implications for Interventions and Mental Health Counseling,” and “Conclusions and Future Efforts,” provide an overview of how Self-Determination Theory provides researchers and practitioners with a framework to improve caregiver relationships. This document will also outline how SDT- informed interventions can assist caregivers by providing them with opportunities to develop supportive social networks. Finally, the authors will summarize these findings, provide additional recommendations for future research, and demonstrate how to develop and implement successful interventions based on Self Determination Theory.

### 1.1 Self-determination theory: a framework for understanding caregiver well-being

Self-Determination Theory (SDT) offers a lean and empirically grounded theory of psychological well-being in any life circumstance, such as health and caregiving. First introduced by Deci and Ryan, SDT suggests that the highest level of functioning relies on the realization of three fundamental psychological needs: autonomy, competence, and relatedness(Ryan & Deci, n.d.). These needs are believed to be universal and needed to maintain motivation, resilience and mental health. In the case of the provision of such needs in social environments, people feel more vital, have an internalized motivation, and well-being, whereas, on the contrary, the frustration of the need is linked to psychological distress, burnout, and ill-being.

Autonomy is the feeling of volition and approval of oneself in an action. People experience their autonomy when they experience decision-making, meaningful contribution to the decision-making process, and when the action is consistent with personal values. In the context of caregiving, autonomy can be impacted depending on the extent to which parents can make informed choices concerning the services, represent their children, and have a sense of personal agency instead of being controlled by medical or institutional structures.

Competence refers to the feeling of efficiency and control over relating to the surrounding. The sense of competence is experienced when people feel that they have the knowledge and abilities that can help them to address issues and attain expected results. In the case of the caregivers, competence is highly associated with caregiving self-efficacy, problem-solving ability, and behavioral and developmental needs management confidence.

Relatedness comprises the sensation of closeness, affiliation, and mutual concern for others. Positive relationships moderate the impact of stress and offer emotional and practical support, contributing to better coping. Relatedness, in the case of mothers of children with ASD, might be influenced by family cohesion, peer support, collaboration with professionals, and stigma or social exclusion.

The mothers with higher self-efficacy and skill mastery on caregiving report lower stress and depressive symptoms in mothers as well as improved family functioning. Psychoeducational programs and parent training has been demonstrated to increase the level of behavioral management and confidence that reflects in improved psychological outcomes (8)

Intervention research implies that parents who believe that they can interpret the needs of their child, introduce strategies, and see improvements get more motivated and less burnt out (9). These results are in line with the SDT conceptualization of competence as a motivator of long-term involvement and well-being. On the other hand, perpetual uncertainty, uneven professional leadership, and complicated service systems can undermine competence perceptions, which can affect emotional exhaustion and withdrawal.

However, contradictions continue to exist. Other training programs can lead to better child outcomes without the need to bring a meaningful change in parental mental health, meaning that the acquisition of skills is not enough without the broader systemic or relational requirements. This implies that competence should not be managed independently of autonomy and relatedness, as it should be supported. There are not many studies that have explicitly investigated competence using SDT frameworks, which is also an important gap in the theory.

Relatedness: Concepts of relatedness hold significant value in the study of social networks. Relatedness, social connectedness. Social networks and relations are important concepts in social network study.

Social connectedness is one of the strongest predictors of maternal well-being in autism caregiving. The mothers often complain of social isolation, a decrease in community life, and stigma about their child's behavior. This form of social exclusion is always linked with prominent levels of anxiety, depression, and low quality of life.

On the other hand, peer support groups, family cohesion, and working relationships with the professionals also have protective roles. The inclusion in parent networks and mutual-aid groups has been associated with better emotional adjustment, problem-solving togetherness, and normalization of the caregiving experiences. The perceived social support is a consistent moderator of the relationship between psychological distress and caregiving demands, which cushions adverse consequences (3)

This observation is in line with the claim of SDT that the relatedness leads to resilience through the delivery of emotional validation and a sense of belonging. Yet, studies tend to approach support as a structural feature (e.g., availability of services) and not as a subjective experience of interrelationship. Further research can also be enriched by separating the existence of supports and the nature of relational experiences, which is one of the main focuses of SDT.

Combined, the data indicates that relatedness can be especially salient in the situation where the demands of caregiving are chronic and socially stigmatized. Strong peer connection and isolation interventions could be significantly important in maternal mental health promotion.

### 1.2 Current research gaps and methodological challenges

Although there is more research about mothers caring for children with ASD than ever before, many limitations still exist in how researchers conceptualize and the methodology of studies relating to this population. A majority of studies examining mothers of children with ASD have focused on issues such as parenting stress, burden of caregivers, anxiety, and depression (10); however, far fewer studies have been conducted from this population from a self-determination theory (SDT). As an illustration (11), showed that autonomy, competence, and relatedness are important factors to consider when studying informal care; however, there has been limited research using SDT as a basis for studying caregivers of children with autism spectrum disorder.

Second, empirical research on maternal well-being in autism caregiving has relied predominantly on cross-sectional designs, limiting causal inference and restricting understanding of dynamic caregiving processes over time (12). Longitudinal investigations remain relatively scarce despite the chronic and evolving nature of autism caregiving, where maternal need fulfillment, motivation, and psychological well-being may shift alongside children's developmental trajectories and changing service demands (13). Furthermore, much of the existing literature employs generalized burden or stress measures rather than Self-Determination Theory (SDT)-congruent constructs that directly assess autonomy, competence, and relatedness among caregivers (14).

Third, there is a lack of contextual diversity of samples. Most research is done in high-income, educated, urban groups; few studies involve low-income, rural, and culturally heterogeneous groups, where the effects of stigma, structural inequalities in service access, and family support are acting differently (rural caregivers are exposed to social isolation and face inadequate access to formal services). This discontinuity limits ecological authenticity and could systematically underrepresent caregivers' demands in low-resource environments (15)

Fourth, earlier research views caregiver support mainly from a structural viewpoint (e.g., service availability, access to formal resources), whereas Self Determination Theory (SDT) views the quality of interpersonal support and the emotional experience of relatedness as being key determinants of well-being (6). Social support and coping strategies can act as mediators in the relationship between systemic stressors and mothers' mental health among mothers of children with autism spectrum disorder (16).

Lastly, there remains a shortage of theory-based intervention trials specifically targeting psychological need support among mothers of children with ASD. Although parent training and psychoeducational interventions may enhance caregiving competence and behavioral management skills, findings regarding maternal mental health outcomes remain inconsistent, suggesting that increased skills alone may not optimize motivation or well-being when autonomy and relatedness needs remain unmet (8).Reviews of psychosocial interventions for parents of children with autism have similarly reported substantial variability in caregiver outcomes and emphasized the need for more robust, theoretically grounded intervention designs (16)

### 1.3 Implications for interventions and mental health practice

The practical implications of SDT applied to interventions and clinical practices include a number of applications. Interventions involving support of autonomy and the method of decision-making through collaboration, goal setting, and respect for caregivers' preferences can be effective in increasing the feeling of volition and control of the challenges of caregiving in mothers. Early caregiver support initiatives integrating adaptable service orientation and culturally informed modules, such as peer-led interventions that tackle barriers to involvement, are plausible and acceptable and could reinforce maternal agency and service involvement in a peer-supported ASD caregiver intervention (17).

Support for building competency should go beyond the primary emphasis on delivering new skills through traditional training methods into implementing strategies that help to strengthen a caregiver's confidence, mastery, and ability to make decisions while providing care each day. The findings of existing research show that caregivers who actively incorporate what they have learned and receive individualized feedback from their peers improve their motivation, self-efficacy, and mental health (18). Despite this evidence, many of the current intervention models emphasize the acquisition of behavioral skills, which limits the ability of an intervention to offer strategies for continuing the development of competency and meeting the psychological needs of caregivers.

In the case of relatedness, isolation and stigma can be directly tackled in the context of interventions that enhance social bonding, such as peer support groups, family systems approach, and community networks. Mothers tend to describe that positive interpersonal contact with other caregivers helps alleviate emotional distress, and positive professional relationships are even more effective in preventing stress. In line with this, relational elements should be incorporated into autism caregiver support (e.g., helping peer groups, family therapy, etc.) as it may enhance mental health improvements that are not about the informational content alone (19).

Clinically speaking, SDT-informed lens adoption can be used to augment screening, referral, and psychosocial support systems. Autonomy support (perceived choice and control), competence beliefs, and social connectedness could be examined by mental health professionals who work with autism caregivers in routine evaluations, and interventions designed to address them would be tailored. Also, systemic barriers, including poor access to services, stigma, and financial burden, are important to the needs support and long-term well-being of caregivers (20).

### 1.4 Future directions

By redefining maternal well-being when providing autism care under SDT, it is apparent that psychological functioning goes beyond burden reduction to self-determination, agency, and resilience. Declarations in current literature highlight the importance of need satisfaction being associated with enhanced motivation and well-being among caregivers in general, but the particular relation of the concept to autism caregiving is immature and still disjointed (21).

To this end, future studies in these areas should focus on longitudinal and mixed-method surveys that would capture changes in autonomy, competence, and relatedness over time, specifically as caregiving needs vary across developmental periods or transition periods of life. The application of validated SDT measures and standard mental health measures will enhance the causal inference and theoretical conformity. Researchers must also aim for a more comprehensive sample that is indicative of socio-economic, cultural, and geographic diversity, thus enhancing generalization and equity in the study of caregivers (22).

Also, the SDT-based intervention research is a significant frontier. Randomized controlled trials, which incorporate need-supportive elements in caregiver training, peer support, and family-centered services, may shed light on how change occurs and also inform scalable models of clinical practice. Not only are behavioral and child outcomes considered by researchers, but caregiver motivational processes and psychological need satisfaction.

Valid policy implications are also essential: the autonomy and relatedness on the structural levels can be reinforced by means of the service systems that augment caregiver choice, improve accessibility, and address stigma. Interprofessional models that can involve caregivers as active participants in care planning and service design can be useful in mitigating systemic obstacles and promoting long-term health.

To conclude, it is true that research and practice should shift towards a deficit-based approach to stress and burden to a need-informed framework of strength-based forecasting of self-determination and psychological resilience. It will do so and offer a more polyphasic, practical sense of maternal well-being in autism caregiving and produce interventions that actually address the psychological needs of caregivers.

## References

[1] American Psychiatric Association. Diagnostic and Statistical Manual of Mental Disorders. 4th ed Washington. DC: American Psychiatric Association (1994).

[2] Van Niekerk K Stancheva V Smith C. Caregiver burden among caregivers of children with autism spectrum disorder. South Afr J Psychiat. (2023) 29:2079. 10.4102/sajpsychiatry.v29i0.2079 PMC10623632 37928940

[3] Cetinbakis G Bastug G Ozel-Kizil ET. Factors contributing to higher caregiving burden in turkish mothers of children with autism spectrum disorders. Int J Dev Disabil. (2020) 66(6):439–47. 10.1080/20473869.2018.1478630 PMC8115497 34141366

[4] Demirpençe Seçinti D Diş D Albayrak ZS Şen E. Depression and parental distress among caregivers of autistic children: a serial mediator analysis in caregivers of autistic children. BMC Psychol. (2024) 12(1):339. 10.1186/s40359-024-01704-x 38858797 PMC11165892

[5] Barry RA Longstreth ME Benson K Cannon CJ Gomez Batista S Slosser Worth A. Testing a self-determination theory perspective of informal caregiving: a preliminary study. Psychol Aging. (2021) 36(7):855–69. 10.1037/pag0000648 34647767

[6] Ryan RM Deci EL. Self-determination theory. In: Maggino F, editor. Encyclopedia of Quality of Life and Well-being Research. Cham: Springer International Publishing (2024). p. 6229–35.

[7] Desimpelaere EN Soenens B Prinzie P Waterschoot J Vansteenkiste M Morbée S. Parents’ stress, parental burnout, and parenting behavior during the COVID-19 pandemic: comparing parents of children with and without complex care needs. J Child Fam Stud. (2023) 32(12):3681–96. 10.1007/s10826-023-02702-0

[8] Iadarola S Pérez-Ramos J Smith T Dozier A. Understanding stress in parents of children with autism spectrum disorder: a focus on under-represented families. Int J Dev Disabil. (2019) 65(1):20–30. 10.1080/20473869.2017.1347228 30873280 PMC6411305

[9] Althoff CE Dammann CP Hope SJ Ausderau KK. Parent-mediated interventions for children with autism spectrum disorder: a systematic review. Am J Occup Ther. (2019) 73(3):7303205010p1–7303205010p13. 10.5014/AJOT.2019.030015 31120831

[10] Karst JS Van Hecke AV. Parent and family impact of autism spectrum disorders: a review and proposed model for intervention evaluation. Clin Child Fam Psychol Rev. (2012) 15(3):247–77. 10.1007/s10567-012-0119-6 22869324

[11] Dombestein H Norheim A Lunde Husebø AM. Understanding informal caregivers’ motivation from the perspective of self-determination theory: an integrative review. Scand J Caring Sci. (2020) 34(2):267–79. 10.1111/scs.12735 31313852

[12] Benson PR Kersh J. Marital quality and psychological adjustment among mothers of children with ASD: cross-sectional and longitudinal relationships. J Autism Dev Disord. (2011) 41(12):1675–85. 10.1007/s10803-011-1198-9 21347614

[13] Totsika V Hastings RP Emerson E Lancaster GA Berridge DM. A population-based investigation of behavioral and emotional problems and maternal mental health: associations with autism spectrum disorder and intellectual disability. J Child Psychol Psychiatry. (2013) 52(1):91–9. 10.1111/j.1469-7610.2010.02295.x 20649912

[14] Dieleman LM Soenens B Vansteenkiste M Prinzie P Laporte N De Pauw SSW. Daily sources of autonomy-supportive and controlling parenting in mothers of children with ASD: the role of child behavior and mothers' psychological needs. J Autism Dev Disord. (2019) 49(2):509–26. 10.1007/s10803-018-3726-3 30145734

[15] Wan L Wu Y Tang Y Guo X Sun L He Q. Pressure and coping strategies of caregivers of children with autism spectrum disorder in rural areas: a qualitative study. Sci Rep. (2026) 16(1):4740. 10.1038/s41598-025-34876-6 41486197 PMC12873353

[16] Khusaifan SJ El Keshky MES. Social support as a protective factor for the well-being of parents of children with autism in Saudi Arabia. J Pediatr Nurs. (2021) 58:e1–7. 10.1016/j.pedn.2020.11.014 33317948

[17] Iadarola S Pellecchia M Stahmer A Lee HS Hauptman L Hassrick EM. Mind the gap: an intervention to support caregivers with a new autism spectrum disorder diagnosis is feasible and acceptable. Pilot Feasib Stud. (2020) 6(1):124. 10.1186/s40814-020-00662-6 PMC7487627 32944273

[18] Martela F Ryan RM. Distinguishing between basic psychological needs and basic wellness enhancers: the case of beneficence as a candidate psychological need. Motiv Emot. (2020) 44(1):116–33. 10.1007/s11031-019-09800-x

[19] Cherewick M Daniel C Shrestha CC Giri P Dukpa C Cruz CM. Psychosocial interventions for autistic children and adolescents delivered by non-specialists in low- and middle-income countries: a scoping review. Front Psychol. (2023) 14:1181976. 10.3389/fpsyg.2023.1181976 37609501 PMC10440606

[20] Uysal G Karakul A Düzkaya DS. Emotions and difficulties experienced by parents of children with autism: a qualitative study. J Eval Clin Pract. (2025) 31(5):e14207. 10.1111/jep.14207 39440802

[21] Lohiya N Srivastava L Lohiya N Kalrao V. Stress among mothers of children with autism Spectrum disorder in comparison to children with & without special health care needs. J Pediatr Rehabil Med. (2023) 16(3):473–81. 10.3233/PRM-220014 36776080

[22] Zijlmans LJM Embregts PJCM Gerits L Bosman AMT Derksen JJL. The effectiveness of staff training focused on increasing emotional intelligence and improving interaction between support staff and clients. J Intellect Disabil Res. (2015) 59(7):599–612. 10.1111/jir.12164 25171725

